# Differential effects of TNF-α and IL-1β on the control of metal metabolism and cadmium-induced cell death in chronic inflammation

**DOI:** 10.1371/journal.pone.0196285

**Published:** 2018-05-16

**Authors:** Paola Bonaventura, Aline Lamboux, Francis Albarède, Pierre Miossec

**Affiliations:** 1 Department of Immunology and Rheumatology, Immunogenomics and Inflammation Research Unit EA 4130, University of Lyon, Edouard Herriot Hospital, Lyon, France; 2 Geology Laboratory–Department of Earth Sciences, Ecole Normale Supérieure de Lyon and CNRS Lyon, France; Universite Paris-Sud, FRANCE

## Abstract

**Objective:**

Interleukin-1-beta (IL-1β) and tumour necrosis factor-alpha (TNF-α) are both monocyte-derived cytokines. Both cytokines have been previously described to exert a role in rheumatoid arthritis (RA) pathogenesis synergizing with other pro-inflammatory mediators, such as interleukin-17 (IL-17) on target cells, for the perpetuation of the inflammatory response (e.g. IL-6 production). In the context of experimental RA, Cd addition has an anti-proliferative and anti-inflammatory effect when associated to IL-17/TNF-α stimulation, due to its accumulation in synoviocytes. The aim of this work was to evaluate if IL-1β interaction with IL-17 also contributes to metal-import mechanisms and its effects on cell viability and inflammation.

**Methods:**

IL-17 and IL-1β were added to synoviocyte cultures with or without exogenous Cd addition (0.1 ppm, 0.89 μM). IL-6 production, Cd import kinetics, gene expression of ZIP-8 importer and metallothioneins (MTs) and cell viability were evaluated by ELISA, inductively-coupled mass spectrometry (ICP-MS), q-RT-PCR and viability assays (neutral red and annexin V) respectively.

**Results:**

IL-17 and IL-1β acted in synergy on synoviocytes to induce IL-6 production similarly to the IL-17/TNF-α combination. Metal import was lower with IL17/ IL-1β in comparison to IL-17/TNF-α exposed-synoviocytes, as the expression of ZIP-8 and MT-1F was less induced. Monocyte and PBMCs exposure to Cd resulted in a reduced production of IL-1β and an increased production of TNF-α and this result was confirmed in co-cultures of synoviocytes and PBMCs. The IL-17/IL-1β combination with Cd slightly reduced cell viability in comparison to the IL-17/TNF-α combination and resulted in a strong induction of IL-6 production.

**Conclusion:**

IL-17/TNF-α combination but not IL-17/IL-1β combination mainly drives the accumulation of Cd in synoviocytes and its effects on cell viability and inflammation.

## Introduction

Interleukin-1-beta (IL-1β) and tumour necrosis factor alpha (TNF-α) are key cytokines produced by monocytes and activated macrophages. Together with lymphocyte-derived cytokines such as interleukin-17 (IL-17) they are the most important mediators of inflammation in the context of rheumatoid arthritis (RA) [1], contributing to chronic inflammation.

IL-1β and TNF-α are both involved in a variety of cellular activities (cell proliferation, differentiation, and cell death) [2,3] but they differ for their structure, the cellular responses they induce and pathways of activation [4]. In particular, IL-1β is activated by the NALP-1 caspase-1 inflammasome cascade and participates in the process of cell pyroptosis, characterized by the release of massive cytokine levels [5]. TNF-induced cell death acts through the caspase-8/caspase-3 pathway, inducing auto-proteolytic activation, leading to apoptosis [6,7].

In various RA models, IL-1β and TNF-α mobilize and activate leukocytes, inducing B, T and natural killer cell proliferation [1]. Th17-cell produced IL-17, in turn, regulates IL-1β and TNF-α production [8,9] sometimes resulting in a synergistic interaction of IL-17 with one or both cytokines to increase the inflammatory response [10,11].

IL-17, TNF-α and IL-1β act synergistically on the transport of divalent metals in human cells, through the regulation of ZnT-Irt (ZIP-importers) and metallothionein (MTs, homeostasis regulators) gene expression [12]. In particular, ZIP-8 importer is highly expressed on fibroblasts and ZnT1 is mainly involved in the membrane export of Zinc (Zn) to extracellular fluid. MTs-1 are necessary for cell growth of human primary cells [13], are responsible for the homeostasis of Zn, and play a detoxifying role against the accumulation of heavy metals such as Cadmium (Cd) inside cells. The net effect in favour of metal import induced by IL-17/TNF-α by in vitro stimulation was used previously used to favour the accumulation of Cd inside synoviocytes. In vivo in an RA animal model, Cd injection in joints induced apoptosis, reduced inflammation, and destruction [14].

The aim of this study was to understand to which extent monocyte-derived cytokine IL-1β, by interacting with IL-17, contributes to changes in metal metabolism of synoviocytes and could contribute to the changes of the inflammatory status and to cell proliferation in our *ex vivo* model and eventually contribute to the anti-inflammatory effect previously demonstrated *in vivo*. Results show the main importance of the IL-17/ TNF-α pathway in Cd-induced cytotoxicity.

## Materials & methods

### Cell culture and activation

Synoviocytes were grown from fresh synovial tissue samples aseptically isolated from RA patients’ joints. The RA patients fulfilled the American College of Rheumatology criteria for RA. All patients signed an informed consent form and the study was approved by the ethics committee of the hospitals of Lyon. All methods were performed in accordance with these guidelines and regulations. Due to the paucity of immune cells in patients undergoing treatment and the difficulty to isolate and use the autologous system for *in vitro* studies, a model using fibroblasts co-cultured with PBMCs was previously developed and used in this study [15].

Synoviocytes were pre-exposed overnight to IL-17A at 50 ng/ml (R&D systems, Minneapolis, MN, USA) and/or IL-1β, at 10 or 100 pg/ml or TNF-α at 500 pg/ml, (both R&D systems) prior to metal exposure, in normal medium (complete DMEM with 10% FBS). The synergistic effect of IL-17 and TNF-α on IL-6 production was used to identify the dose of IL-1β to be used in co-exposure with IL-17. The day after, Cd was dissolved in Nitric acid 5% for a first dilution (1:10) and was secondly diluted 1:100 (to reach 0.1 part per million (ppm), corresponding to Cd 0.89 μM) directly in the culture medium, in the presence or not of the cytokines previously added.

Peripheral blood mononuclear cells (PBMCs) and monocytes, obtained by adhesion, were activated or not with Phytohemagglutinin (PHA, 5 μg/ml) before being exposed to Cd, according to results showing PHA inducing similar levels of pro-inflammatory cytokines as more specific LPS (10 ng/ml) (data not shown) and to maintain the same stimulus as for total PBMCs. Co-cultured PBMCs (stimulated or not with PHA) with adherent synoviocytes represent our *ex-vivo* model to mimic the inflammatory situation in joints and helping in the understanding of the role of IL-1β in the transport of Cd.

### IL-6, IL-1β and TNF production measurement by ELISA

The synergistic effect of IL-17 (50 ng/ml) and TNF-α (500 pg/ml) on the production of IL-6 by synoviocytes was already evaluated in previous papers [11,12]. Here a dose-response curve was established, comparing the known effect of IL-17/TNF combination to the one of IL-17 in association with IL-1β (10 and 100 pg/ml). IL-1β was first used alone or in association with IL-17 to verify the synergistic effect.

IL-6 production was quantified in synoviocyte and co-culture supernatants of day 5 by standard ELISA techniques (R&D system, San Diego, CA, USA). IL-1β and TNF-α production was measured in PBMC and monocyte cultures or in co-cultures in the presence or not of Cd. Absorbance at 450 nm was measured using the VICTOR X4 plate reader and results were obtained by subtracting the background read at 540 nm and compared to the regression curve obtained by standards of R&D system kit following manufacturer instructions.

### Kinetics and cell content of metals by ICP-MS

To analyse Cd kinetics and cell content, 2 ml of supernatant were collected at 6, 24, 48 hours. Cells were than washed with PBS and fresh complete DMEM was substituted to the Cd-enriched medium, to study the possible exit of Cd from cells. Two ml of medium were collected immediately after the wash and at 120 hours, while cells were collected and counted at the end-point (120 hours). All the samples were then mineralized with HNO_3_ 15N plus H_2_O_2_ (30%) at 100°C. Prior the analysis, the mineralized samples were re-dissolved in a 5% HNO_3_ solution in deionized water in an ultrasonic bath. Metal ions in the samples were then measured on a single collector ICP-MS platform ELEMENT 2 (Thermo Finnigan, Ringoes, NJ, USA) which allows metals to be measured at concentrations levels as low as 10–12 units, i.e. in the part per trillion (ppt) range. The detector receives an ion signal proportional to the metal concentration.

### Quantification of the gene expression of Zn transporters by quantitative real-time PCR

RA synoviocytes were plated at a density of 2,5x10^5^ cells/cm^2^ in 12-well plates and were then exposed or not to cytokines overnight followed or not by Cd exposure for 6 hours. After 6 hours of treatment, total RNA was extracted using the RNeasy Mini Kit (Qiagen, Hilden, DE) and quantified with the Quant-it kit assay (Invitrogen by Thermo Fisher Scientific, Grand Island, NY, USA) following manufacturer’s instructions. cDNA was synthesized using the QuantiTect reverse transcription kit (Qiagen) according to the manufacturer’s instructions. SYBR green-based real-time qRT-PCRs were performed on the CFX96 Real-Time PCR Detection System (BioRad, Hercules, CA, USA) using the QuantiFast SYBR green kit and QuantiTect primers (Qiagen). Cycle threshold values were normalized with respect to the endogenous control gene glyceraldehyde 3-phosphate dehydrogenase (GAPDH). The relative expression of the genes in treated cells versus control cells was determined using the comparative threshold cycle method as described by the manufacturer.

### Cell viability quantification by neutral red assay and annexin V staining

The neutral red assay determines the accumulation of the neutral red dye in the lysosomes of viable cells as described by Borenfreund and Puerner [16], while annexin V staining is used to stain cell undergoing apoptosis. Synoviocytes were plated in duplicates at a density of 10^4^ cells/cm^2^ in 96-well plates. After optional exposure to cytokines, metals (Cd alone or Cd plus Zn) for 5 days annexin V staining and neutral red assay were performed. For the neutral red assay cells were incubated for 150 min with 80 μg/ml of neutral red dye 0.33% (Sigma-Aldrich, St. Louis, MO, USA) at pH 6.5 in serum-free DMEM. Cells were then washed with PBS followed by 10 min incubation in 200 μl of elution medium (ethanol/acetone, 50%/1% in deionized water). Absorbance at 540 nm was measured using VICTOR X4 plate reader (Perkin Elmer, Waltham, MA, USA) and results were obtained by subtracting the background read at 690 nm and the 540 nm absorbance in medium without cells.

Annexin V staining was performed on trypsinized cells suspended in the annexin V buffer. Annexin V APC Ab was added in a dilution 1/200 per well (Ab and buffer provided by BD biosciences, San José, CA, USA). After 15 minutes of incubation at room temperature in the dark, samples were analysed by FACS Navios (Beckman Coulter, Brea, CA, USA).

## Results

### Low IL-1β concentration synergizes with IL-17 stimulating IL-6 production by synoviocytes similarly to the IL-17 TNF combination

**Fig 1:**
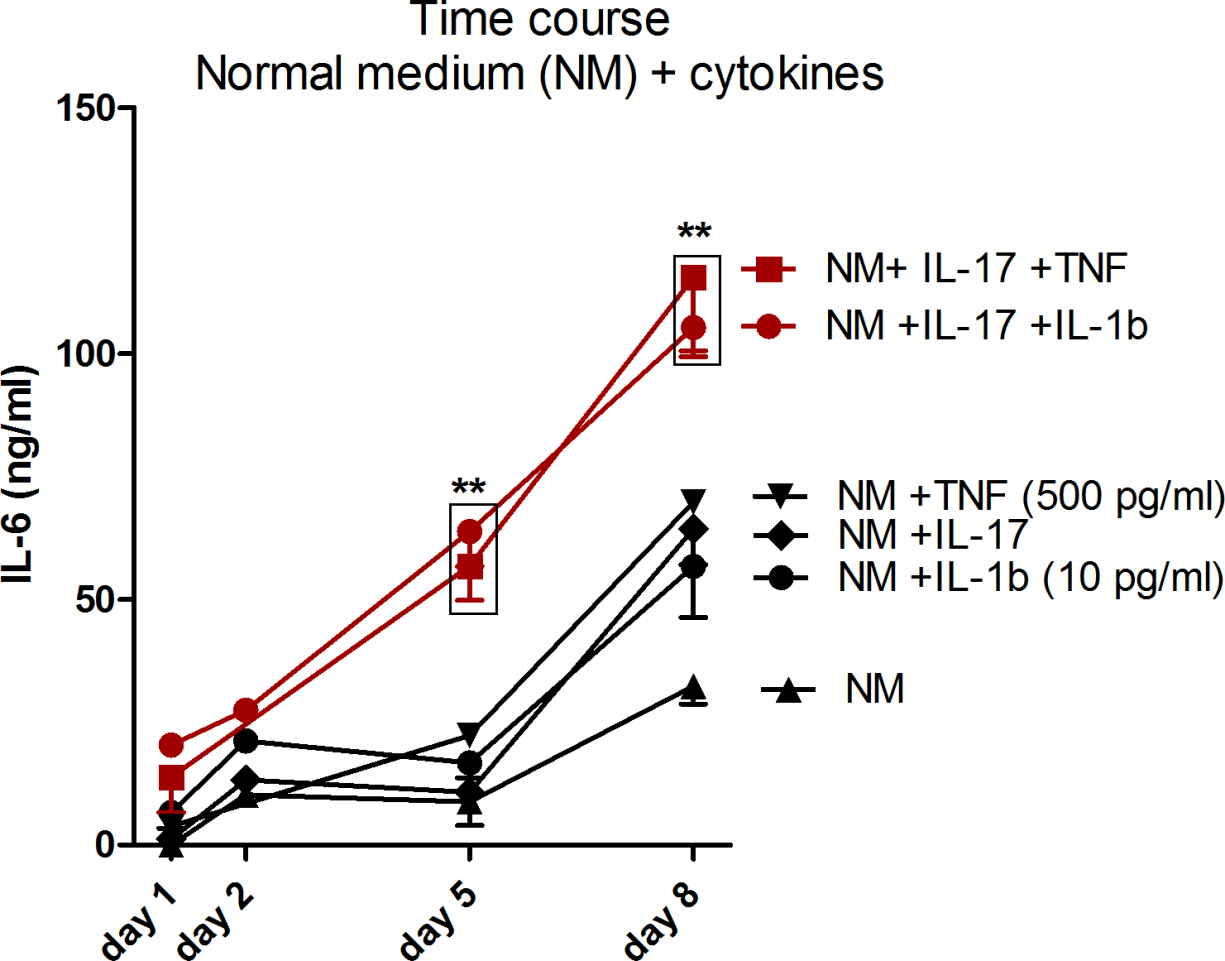
IL-1β synergizes with IL-17 to produce IL-6, independently from Cd exposure. Time course of IL-6 production in supernatants of synoviocytes exposed to cytokine combinations. Red lines indicate the chosen condition to perform the next experiments. Data are represented as mean ± SEM of at least three independent experiments; * shows differences between non-inflammatory and inflammatory conditions, # shows differences due to Cd addition; */# p<0.05, **/##p<0.01.

The ability of IL-1β to synergize with IL-17 was tested on IL-6 production by synoviocytes. At this time-point, synoviocytes not stimulated by pro-inflammatory cytokines produced 14.36 ± 6.75 ng/ml of IL-6, those stimulated with IL-17 alone 10.79 ± 1.45 ng/ml and those stimulated with IL-1β alone 16.76 ± 3.21 ng/ml. IL-17 synergized with IL-1β at 10 pg/ml at day 5 producing 63.78 ± 9.77 ng/ml of IL-6 (p<0.01 in comparison to control at day 5, Fig 1) and this value was very comparable to the 56.76 ± 10.23 ng/ml of IL-6 produced at the same time-point by synoviocytes after IL-17 plus TNF-stimulation. IL-1β used alone at 100 pg/ml resulted in a far higher production of IL-6 in comparison to other cytokines alone (58.53 ± 18.09 ng/ml) reducing the effect of the synergy with IL-17 (83.27 ± 18.09 ng/ml, data not shown). 10 pg/ml was then chosen as the dose to be used in the next experiments to compare the effect of the IL-17/IL-1β to the IL-17/TNF combination. After 8 days, the synergistic effect of cytokines was still measured in both conditions (IL-17 in association with 10 pg/ml of IL-1β or with 500 pg/ml of TNF-α).

### IL-17/TNF-α but not IL-17/IL-1β combination drives Cd accumulation in synoviocytes

**Fig 2:**
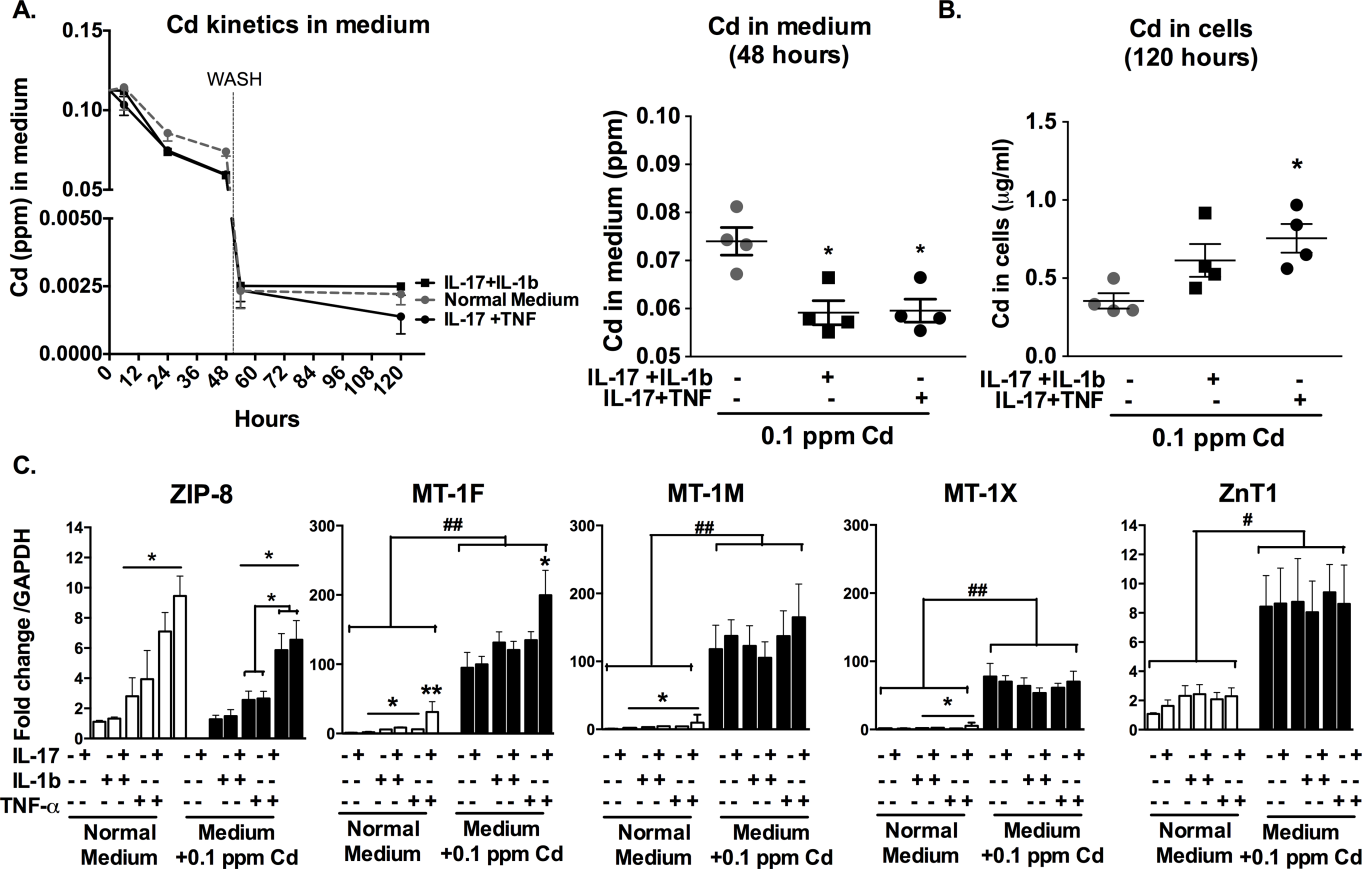
Cd slightly accumulates in synoviocytes exposed to IL-17/IL-1β, through the reduced expression of ZIP-8 and MT-1F. **A.** Cd concentration in medium as function of time and after 48-hour exposure. Gray dashed line: control experiment with no cytokine addition. Vertical dashed line at 48 hours: wash. **B.** Cd concentration in synoviocytes after 48-hour exposure to cytokines followed by wash and 72 hours in normal medium. Measured performed at 120 hours. **C.** Gene expression of Cd transporters in comparison to GAPDH. Black histograms: exposure to Cd. Data are represented as mean ± SEM of at least three independent experiments; * shows differences between non-inflammatory and inflammatory conditions, *p<0.05.

The kinetics of Cd in the culture medium was analysed after exposure to IL-17 (50 ng/ml) plus IL-1β at the selected concentration of 10 pg/ml and compared to the kinetics of Cd in the presence of IL-17 and TNF-α (500 pg/ml) combination, resulted in an equal production of IL-6. For both conditions, Cd uptake started 12 hours after Cd-exposure. After 48 hours, Cd content in the medium was decreased in cytokine-stimulated conditions in comparison to the control (0.059 ± 0.003 ppm in cytokine stimulated conditions vs. 0.074 ± 0.002 ppm in the control, p<0.05) **(Fig 2A)**. After 48 hours, a wash with new medium without Cd enrichment was performed. Excess of Cd were then removed and its level in the fresh medium was similar among the different conditions (~ 0.0025 ppm). Synoviocytes previously exposed to IL-17 and TNF-α continued to absorb residual Cd after the wash, as Cd concentration in the medium was still reduced at 120 hours (~ 0.0015 ppm). Conversely, the concentration of Cd in the medium did not vary between the two time-points when cells were not pre-exposed to cytokines or were exposed to IL-17 and IL-1β combination **(Fig 2A)**.

Cd cell content at the end-point (120 hours) was increased in cells exposed to IL-17 and TNF- α in comparison to control (0.76 ± 0.09 μg/ml vs. 0.35 ± 0.05 μg/ml respectively, p<0.05). Conversely the exposure to IL-17 and IL-1β combination did not increase significantly Cd content in cells (0.61 ± 0.10 μg/ml) **(Fig 2B)**.

Inflammation has a pivotal role in the regulation of the expression of ZIP-8 importer [12]. Interestingly, IL-17/IL-1β combination enhanced the expression of the importer (2–4 fold) with a weaker effect in comparison to IL-17 plus TNF-α combination (6–10 fold). This difference was especially exacerbated in the presence of Cd, where ZIP-8/GAPDH fold change in comparison to control was 6.6 ± 0.9 (p< 0.05, **Fig 2C)** in cells stimulated with IL-17/TNF combination vs. only 2.6 ± 0.3 for cells stimulated with IL-17/IL-1β combination. This result showed the main importance of the IL-17/TNF pathway in the regulation of Cd entry through ZIP-8.

MTs are the regulators of metal homeostasis in cells, acting as detoxifying agents against Cd toxicity [17]. MT-1s expression is mainly up-regulated by Cd exposure [14]. The expression of the MT isoforms MT-1M and MT-1X was up-regulated by exposure to cytokines (IL-17, IL-1β and TNF-α alone or in combination with a similar effect). The addition of IL-17 plus TNF-α induced a fold change increase of MT-1M in comparison to control of 9.8 ± 7.4 (p<0.05), compared to 4.5 ± 1.0 (p<0.05) with the addition of IL-17 plus IL-1β combination. For MT 1-X, the IL-17/TNF-α combination induced a fold change of 5.9 ± 2.1 (p<0.05), compared to 2.8 ± 0.6 (p<0.05) with the IL-17/IL-1β combination in comparison to the control condition. MT-1F expression was particularly enhanced by the IL-17/TNF-α combination, increasing 30-fold over the control in the absence of Cd (p<0.01). In the presence of Cd, MT-1s expression was highly enhanced (to the lesser extent 80-fold over control). MT-1F expression increased 90-fold over the control situation in the presence of Cd alone and its expression was doubled in the presence of both Cd and IL-17/TNF-α combination (p<0.05, in comparison to the condition treated with Cd only), but not in the presence of IL-17/IL-1β combination **(Fig 2C)**.

The ZnT1 exporter expression was not modified by IL-17 in combination with TNF-α or IL-1β. Conversely, Cd induced the expression of the exporter 8-fold over the control **(**p<0.05, **Fig 2C)**.

### Cd shifts the monocyte-derived cytokine production through the TNF-α pathway

**Fig 3:**
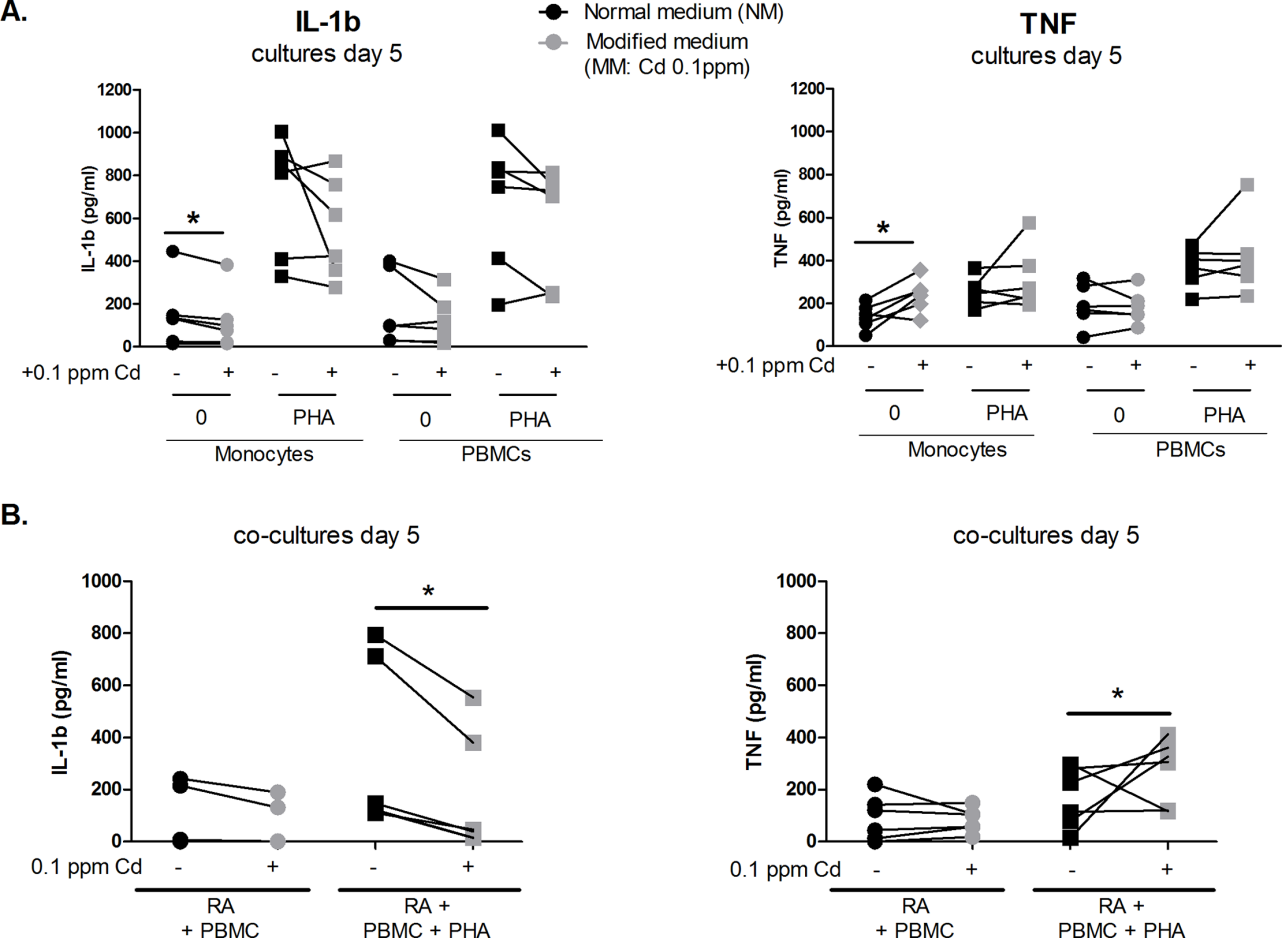
Cd shifts the monocyte-derived cytokine production through the TNF pathway. IL-1β and TNF-α production were measured after 5 days of exposure to Cd **A.** In single-cultured monocytes and PBMCs, activated or not with PHA **B.** In co-cultures of RA synoviocytes and PBMCs activated or not with PHA. Data are represented as mean ± SEM of at least three independent experiments; * shows differences between non-inflammatory and inflammatory conditions, *p<0.05. Linked points represent cells from the same subject.

The effect of Cd was tested in cultures of monocytes and PBMCs, and in co-cultures of synoviocytes with PBMCs to understand to which extent Cd could modify monocyte-derived cytokine production. In single-cell cultures (monocytes or PBMCs alone), a tendency to a decreased production of IL-1β (10.2 to 27.1% depending on culture conditions, with or without PHA) and an increased production of TNF-α (4 to 38% depending on culture conditions, with or without PHA) was measured. The pro-inflammatory cytokine balance was substantially modified in the cultures of unstimulated monocytes, resulting in a 20.1%-reduction of IL-1β and a 38%-increased-TNF-α production with Cd treatment, in comparison to monocytes cultured in normal medium (p<0.05) (**Fig 3A**).

Co-cultures of synoviocytes and PBMCs were performed to mimic the effect of the immune system on inflammatory synoviocytes and to understand the final effect on target cells. A strong reduction of IL-1β was measured after a 5-day-exposure to Cd in co-cultures of synoviocytes with PHA-activated PBMCs, resulting in a mean reduction from 344.2 ± 133.2 pg/ml to 174.9 ± 95.3 pg/ml. Conversely TNF-α mean production was increased from 168.6 ± 47.4 pg/ml to 273.7 pg/ml ± 51.3 (p<0.05, **Fig 3B**).

In conclusion, in the cell interactions as found in arthritis, Cd induced an increased TNF-α/IL-1β ratio, resulting in an active import and storage of Cd inside the cell.

### Cd addition in the presence of IL-1β increases IL-6 production

The effect of Cd could not only be restricted to the changes in TNF-α /IL-1β balance but could modify cell viability and IL-6 production, the most abundantly pro-inflammatory cytokine produced by synoviocytes. As already shown, the IL-17/TNF combination with Cd induced massive synoviocyte apoptosis, resulting in a strong reduction of IL-6 production [14].

**Fig 4:**
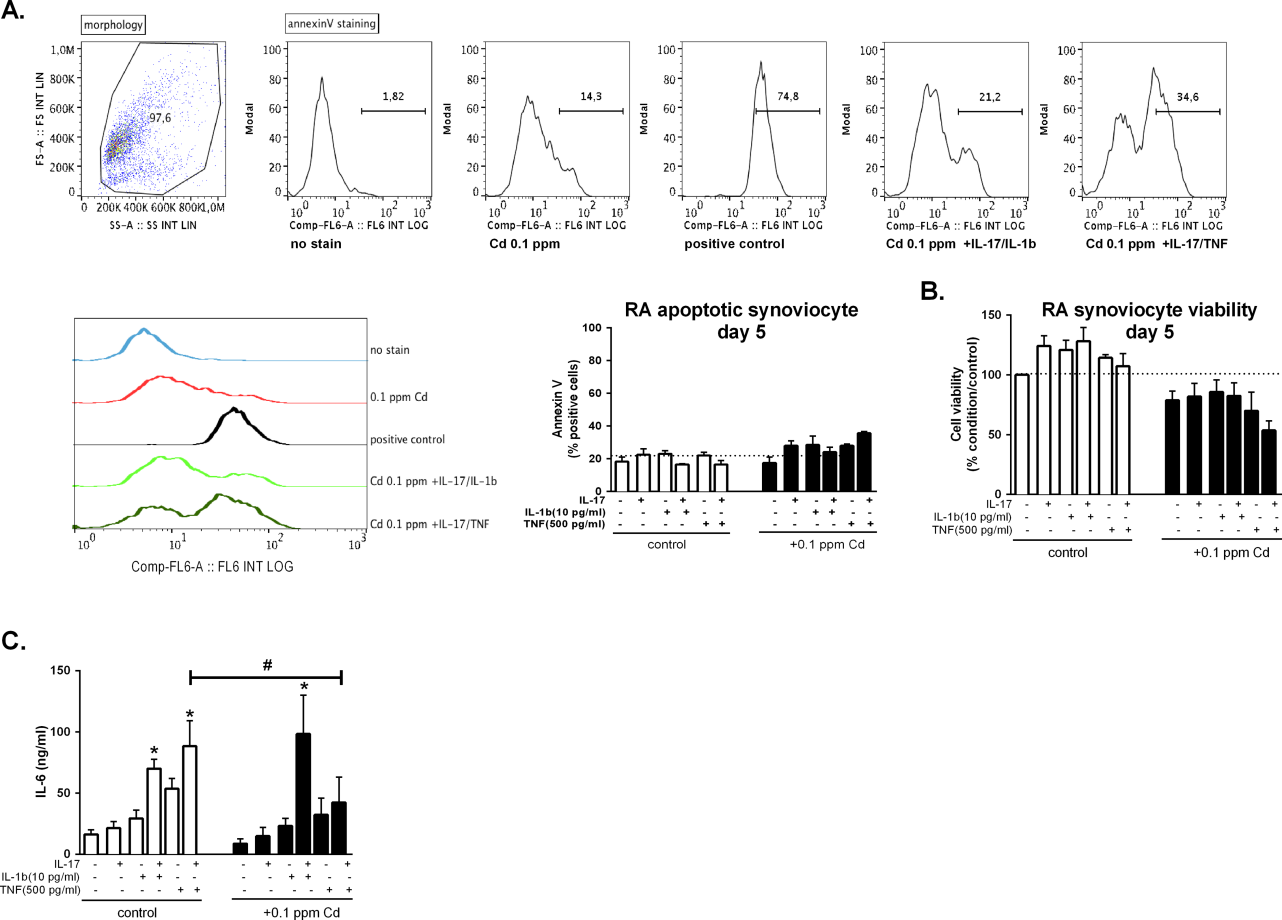
Cd addition in the presence of IL-1β increases IL-6 production. **A.** Annexin V staining of Cd synoviocytes stimulated or not with IL-17 TNF-α and IL-1β and their combination in the presence or not of Cd after 5 days. Dot plot of morphology and histrograms of annexin V staining representative of the experimental conditions (without stain, Cd 0.1 ppm, positive control, 0.1 ppm +IL-17/IL-1 and 0.1 ppm +IL-17/TNF) and graph representing all experimental conditions. **B.** Neutral red assay of synoviocytes stimulated or not with IL-17 TNF-α and IL-1β and their combination in the presence or not of Cd after 5 days. Dashed line in line with 100% viability. **C.** IL-6 production in supernatants of synoviocytes previously exposed to cytokine combinations and to 0.1 ppm of Cd (0.89 μM) for 5 days. Data are represented as mean ± SEM of at least three independent experiments; * shows differences between non-inflammatory and inflammatory conditions, # shows differences due to Cd addition; */# p<0.05.

Apoptosis and cell viability were measured in synoviocytes stimulated with IL-17, TNF and IL-1β alone or in combination and further exposed or not to 0.1ppm (0.89 μM) of Cd after 5 days of culture. Apoptosis was slightly induced by the addition of 0.1 ppm of Cd in the medium while the addition of both Cd and cytokines increased apoptosis. Nevertheless the induction of apoptosis after 5 days was stronger in cells cultured with IL-17/TNF (~35% of cells) and Cd than in other conditions. IL-17/IL-1 combination with Cd induced apoptosis in ~25% of cultured cells (**Fig 1A**) Cell viability (considered as the percentage of viable cells in comparison to control) was slightly increased by cytokine addition (from 3.8% to 28.1%, depending on culture conditions), in line with the anti-apoptotic effect of pro-inflammatory cytokines on synoviocytes [18]. Conversely, in the presence of Cd, cell viability was reduced by 21% in comparison to control and of a further 25% in the presence of Cd plus IL-17/TNF combination. The combination of Cd with IL-17/IL-1β did not decrease cell viability in comparison to control (**Fig 4B**).

Supernatants from the same experiments were collected at day 5 and IL-6 production was measured. IL-6 was reduced in the presence of Cd (0.1 ppm, 0.89 μM) from 88.4 ± 20.7 ng/ml to 42.3 ± 20.7 ng/l ml (p<0.05) when cells are previously stimulated with the combination of IL-17 and TNF-α, as previously demonstrated [14]. Conversely the IL-17/IL-1β combination, in the presence of Cd, induced IL-6 production highly over the Cd-only treated condition (8.7 ± 4.0 ng/ml) and even over the Cd-untreated condition stimulated with the same cytokines (69.9 ng/ml ± 7.9), with a production of IL-6 of 98.4 ± 31.7 ng/ml (p<0.05) (**Fig 4C**)

## Discussion

The close connection between metal metabolism and inflammation [19] has been the focus of our previous research on IL-17/TNF-α inducing metal (Zn and Cd) accumulation in target cells [12,14]. Experiments were performed on synoviocytes derived from RA patients, a model in which the involvement of pro-inflammatory cytokines IL-17, TNF-α and IL-1β is well known [20,21]. As IL-1β actively contributes to enhance metal import in different cell types [22,23], the aim of this study was to evaluate the potential role of IL-1β, alone or in combination with IL-17, on Cd metabolism and Cd-induced cell death, compared to that of TNF-α.

As already described by Chabaud et al. [10], there is a synergistic effect between IL-17 and IL-1β after 5 days, when IL-1β is used at very low concentrations (10 pg/ml) [24]. The first important result shows that a very low concentration of IL-1β (10 pg/ml) compared to TNF-α (500 pg/ml) synergizes with IL-17 to induce IL-6 production by synoviocytes. This result implies the possible involvement of IL-1β in the regulation of metal transport mechanisms as already demonstrated with TNF-α.

Kinetics experiment showed a lower rate of Cd uptake and accumulation in synoviocytes exposed to the IL-17/IL-1β combination, in comparison to the IL-17/TNF-α combination. This result was due to a differential regulation of Cd transporters, in particular ZIP-8 and MT-1F. More precisely, ZIP-8 was not highly regulated by IL-1β, alone or in combination with IL-17, but rather with the IL-17/TNF combination. This difference is not altered in the presence of Cd. MT-1F is highly expressed in the in the presence of IL-17/TNF-α combination, independently from Cd addition. This showed a crucial importance of IL-17/TNF synergy for the enhanced expression of MTs, the homeostasis regulators of metals in the cells. Differently, MT-1F expression in synoviocytes exposed to IL-17/IL-1β combination in the presence of Cd was low, reflecting the effect of Cd alone. While MTs transcription primarily respond to the Cd intracellular content, as previously shown in other cell types [25,26], IL-17/IL-1β combination did not affect their expression.

As TNF-α and IL-1β are both principally produced by activated monocytes/macrophages at the RA site of inflammation [27], cytokine measurement in supernatants of single cultures of monocytes or PBMCs was performed in the presence of Cd, as no information is known on Cd modulatory effect on monocyte derived cytokines. Results showed a tendency to a decreased production of IL-1β correlating with an increased production of TNF-α in single cultures exposed to Cd for 5 days. Co-cultures of synoviocytes and PBMCs were then used as an artificial *ex-vivo* model to obtain information on how Cd could possibly modify secreted levels of pro-inflammatory cytokines from both synoviocytes and PBMCs. In this context, Cd reduced IL-1β and IL-6 production but not TNF.

Taken together these results show a major ability of Cd to reduce IL-1β in comparison to TNF and this could partially explains what previously found *in-vivo* in the rat model where Cd accumulation in synoviocytes was mainly driven by the IL-17/TNF-α pathway, which is not compromised by Cd addition [14].

Experiments were next performed to study the effect of IL-17/IL-1β exposure on cell viability and IL-6 production. Cell viability was less modified by the IL-17/IL-1β than with the IL-17/TNF stimulation in the presence of Cd, but the production of IL-6 was highly increased. IL-17 and TNF-α exposure induced an increased MTs expression and Cd uptake in synoviocytes, resulting in reduced cell viability and decreased IL-6 production. The results previously obtained *in vivo* clearly showed a the reduction of inflammation in rat joints after Cd injection [14], implying a larger contribution of the IL-17/TNF upon the IL-17/IL-1β pathway. The low rate of cell death with Cd plus IL-17/IL-1β can be considered a consequence of the reduced import of Cd, but the high production of IL-6 could also be explained by the induction of a different type of cell death (pyroptosis with IL-1β versus apoptosis with TNF) in a lower number of cells. IL-6 overexpression after Cd plus IL-17/IL-1β exposure could in turn have a negative impact on the import of Cd.

In conclusion, IL-1β synergizes with IL-17 to increase synoviocyte IL-6 production, as well as with the IL-17/TNF combination. Cd transporters were differently regulated by IL-17/IL-1β combination, with a lower increase of Cd importer ZIP-8 and the homeostasis regulator MT-1F. This effect results in a reduced accumulation of Cd inside the cells and a reduced activation of the proapoptotic pathway, with a massive production of IL-6. As the intra-articular Cd injection in rat-joints strongly reduced inflammation and the related bone destruction, our experiments suggest the dominant contribution of the IL-17/TNF-α pathway over the IL-17/IL-1β in Cd transport in the context of chronic inflammation.
